# Brains and Railway Accidents

**Published:** 1900-07-07

**Authors:** 


The Hospital

A Journal of

The Medical Sciences and Hospital Administration.

Vol. XXVIII.—No. 719.] Edited by Sir Henry Burdett, K.C.B., and Solomon C. Smith, M.D., M.R.C.P. [July 7, 1900.

### Brains and Railway Accidents.

The evidence given in regard to the cause of the unfortunate accident at Slough is not only of importance as showing how such accidents happen, but is of much interest as suggesting points in psychology and pathology which are apt to be overlooked by some who seem to think that the recurrence of such catastrophes can be prevented by the simple plan of placing an extra man upon the footplate. At first sight it may seem that, as the collision clearly happened in consequence of the driver failing to take note of the signals, which confessedly were against him, the simple and easy cure for such a thing is to have an extra man to do nothing else except look out for signals. But it is clear from the evidence that in this particular case the man who failed was actually at the moment doing nothing else but watching for signals, that, in fact, he was doing at the moment exactly what the suggested third man would have been doing, and that the cause of the disaster was not the lack of a look-out, but the failure of the man engaged upon it to see what he was on the watch for. We take it that the teaching of this accident is the exact opposite of what has been proposed. What is impressed upon us by it is the perfectly well-known fact that the complex inter-relationship between different parts of the nervous system, the sum total of which is consciousness, is liable to momentary breaks at one link or another; and that, although when these breaks are well-marked or prolonged we regard them as pathological, they nevertheless occur among many who, both physically and mentally, would properly be classed as healthy men. What is new is that under modern conditions the occurrence of such a momentary dreamy state may land a man in difficulties which, as in the case before us, may mean death and injury to many. The typical example of such breaks in consciousness is, of course, to be found in certain minor forms of epilepsy; but there are many other conditions, notably toxic conditions, in which although consciousness may not be abolished it may require a much stronger stimulus than usual to bring it into full receptiveness. Such a condition is that of alcoholism, but there are many toxins, some brewed within the economy, by which the same effect may be produced, and the difficulty of excluding them is practically insuperable. Here was a man with a good record, who had been for years at the work, who had carried an enormous number of passengers in safety, who could show that he had had no alcohol, who was in good health, who had no home trouble to worry him, and who could have no interest or motive in doing such a mad thing as over-running the signals, yet, while standing on the watch, he allows his train to run past two signals set against him. He asserts that he never saw them, and says that he "must have lost himself," which probably exactly expresses what happened. Out of this dreamy state, however, he was at once aroused by his mate's exclamation "Whoa!"—so suddenly, indeed, that the hands of the two men met on the regulator as each seized it to shut off steam. How are we to guard against such an occurrence? Certainly not by putting on an extra look-out. The chances are that a man with a variety of things to do will be far less liable to go into a dreamy state than one who is set to hypnotise himself by staring at signals only.

Protection against the recurrence of such accidents must then be sought not in the employment of still more brains, each as fallible as its predecessor, but in the introduction of improved mechanical appliances by which it shall be impossible for a train to over-run a signal. Such appliances have been available for years past, and although at the time of their first introduction there were probably good reasons for not having recourse to them, the whole problem is now altered by the increasing speed at which trains are run, and by the greatly increased crowding of the lines.

The present system of signalling on railways is essentially defective in that its efficiency depends not only upon brains, which are liable to fail, but upon climatic conditions which are constantly varying; that the signals are entirely different by day and by night; and that the occurrence of even a very moderate degree of fog dislocates the whole arrangement. It ought not to be beyond the reach of human ingenuity to devise a plan by which the signal should be shown upon the engine in such form that it should be unmistakeable, or even by which the brake should be applied or the steam cut off automatically. Such things have, we believe, been in the market for thirty or forty years, most of the patents must have run out, and the field is open for inventors. What is certain is if any improvement is to be made it must be on the side of the machine rather than of the man. The human brain must always break down now and again, and there is but little reason for believing that the brains now being manufactured in our Board Schools will be of any more stable type than those produced in preceding generations. Mechanical appliances we may perhaps improve, brains we cannot; and experience has shown that whatever care we take in selecting them they will fail now and then.

